# Evaluation of Implementation of Massachusetts Sports Concussion Regulations: Results of Focus Groups with Athletic Directors

**DOI:** 10.7759/cureus.7691

**Published:** 2020-04-16

**Authors:** Kathleen O'Hara, Julia Campbell, Alcy Torres, Jonathan Olshaker, Jonathan Howland

**Affiliations:** 1 Emergency Medicine, Boston Medical Center, Boston, USA; 2 Pediatric Neurology, Boston University School of Medicine, Boston, USA

**Keywords:** concussion, mild traumatic brain injury, student athletes, athletic directors, focus groups

## Abstract

In 2018, the Massachusetts Department of Public Health (MDPH) conducted focus groups with athletic directors (ADs) from Massachusetts middle and high schools to assess the implementation of legislated regulations relative to the management of concussion (mild traumatic brain injuries; mTBI) among students engaged in extracurricular sports. Two tape-recorded focus groups were conducted with a facilitator. Lists of themes were synthesized by investigators. Overall, participating ADs expressed that the law and accompanying regulations were necessary and important for protecting student athletes, despite some burdensome aspects of implementation. Emerging themes included support for the law, some implementation problems, impact on workload, and recommendations for improving mandated procedures. ADs assume an important role in the management of middle and high school students’ mTBI when given the authority to do so through legislation and regulation. Nonetheless, challenges to the daily application of legislated protocols exist and should continue to be evaluated.

## Introduction

Sports and recreation-related traumatic brain injuries (TBIs) are some of the most common injuries incurred among children and adolescents aged 17 years and younger in the United States; approximately 1.1 to 1.9 million incidents occur annually [1-2]. The Centers for Disease Control and Prevention (CDC) estimate that between 2010 and 2016, approximately two million children aged 18 years and younger sought care for TBIs in emergency departments [3]. The highest rates of emergency room visits for TBIs are among males for head injuries sustained in contact sports including football, basketball, hockey, lacrosse, wrestling, and soccer [3]. TBIs, including concussions (mild TBIs [mTBIs]), can cause serious emotional, physiologic and cognitive problems that range in severity depending on the extent of the injury. Potential consequences of mTBI include attention and memory deficits, impaired motor function, impaired or complete loss of hearing or vision, and behavioral issues, such as diminished emotional regulation, depression, anxiety, aggression, and/or personality changes [4].

All US states currently have legislation relative to managing mTBI in student athletes, and three states have laws that address mTBI among all students, regardless of whether their head injury occurred during athletics or some other activity [5]. Massachusetts (MA) was an early adopter of sports concussion legislation passing Chapter 166, An Act Relative to Safety Regulations for School Athletic Programs in 2010. In 2011, the Massachusetts Department of Public Health (MDPH) issued regulations pursuant to this legislation. The regulations provide standardized procedures for all stakeholders involved in preventing, training, and managing mTBI for students sustaining head injuries resulting from extracurricular athletics [6]. All public and private middle and high schools that are participants of the Massachusetts Interscholastic Athletic Association (MIAA) are subject to these regulations. This includes most of the schools in the state that have a sports program. The regulations mandate the roles and responsibilities of School Nurses (SNs), Athletic Trainers (ATs), Athletic Directors (ADs), coaches, medical providers, students, and parents in managing return-to-play for students with mTBI resulting from participation in extracurricular sports. Responsibilities include annual training, using standardized forms for pre-sports participation, reporting head injury, developing a plan for return-to-school, and ensuring medical clearance for return-to-play [6].

The MDPH has conducted or sponsored a series of program evaluations to assess the implementation of these regulations. Methods include process measurement, such as the quality and quantity of mandatory reporting of mTBI events, focus groups with stakeholders, and stakeholder surveys. In 2013, the MDPH conducted analyses of annual mandatory school reporting of head injuries, a review of policies from a convenience sample of schools, and an analysis of statewide trends in the emergency department (ED) visits for sports-related TBI [7].

In addition to these initiatives, the MDPH engaged the Injury Prevention Center (IPC) at Boston Medical Center to conduct a series of focus groups and surveys with stakeholders about implementing sports concussion regulations. The objectives of these studies were to gain insight into how the law has affected concussion management in schools, and what problem areas exist or have developed in response to the new legislation.

In 2015, the MDPH conducted focus groups with SNs and ATs from MA middle and high schools to assess implementation of legislated regulations [6,8]. Four tape-recorded focus groups were conducted by experienced facilitators. Lists of themes were synthesized by investigators for each focus group. Participating SNs and ATs supported the sports concussion legislation, endorsed that implementation had gone well, indicated that the law empowered them in managing return-to-school/play for students with concussion, and experienced support from their school administrators. Some SNs reported that they had applied relevant procedures to all students with head injuries, regardless of how or where the injury occurred. Despite a generally high degree of support for the law, SNs and ATs felt that school teachers, counselors, athletic coaches, and healthcare providers could use more education and training regarding sports concussion management [6]. A subsequent survey of SNs confirmed the focus group finding that almost all SNs had generalized aspects of the sports concussion regulations to all students, regardless of how or where their head injury occurred [8].

ADs are responsible for overseeing athletic programs at schools [9]. The duties of ADs include, but are not limited to: hiring and supervising staff and coaches; managing team events, scheduling, operations, and finances; and keeping track of policy changes implemented by the school board or the state. MA sports concussion legislation requires ADs to conduct a number of trainings and reviews throughout the year, and ensure regulation compliance among parents, school staff, coaches and student athletes [9].

ADs are significant stakeholders in the identification and management of concussion among student athletes, and thus their viewpoint is important to a full understanding of the implementation of sports concussion regulations. Accordingly, focus groups were also conducted with ADs to assess their experiences of implementation of sports concussion regulations. The results of these focus groups are reported herein.

## Materials and methods

Development and implementation of focus groups

An evaluation team was convened that included investigators from the IPC and MDPH. This team conducted two focus groups with ADs in March, 2018 at the Massachusetts Secondary School Athletic Director’s Association (MSSADA) annual meeting. Participants were recruited by email invitations sent to all members of the MSSADA. The focus group facilitator for both groups was a licensed AT. Investigators (JH, KO) took notes throughout the group discussion. Additionally, both focus groups were audiotape recorded.

The introduction was scripted by the evaluation team and presented by the facilitator at the beginning of each focus group session. The facilitator provided each participant with a copy of the consent form, which explained: the purpose of the study; that participants would complete a short questionnaire at the beginning of the session; the rights of participants as research subjects (including that they were free to withdraw at any time); and confidentiality procedures. To safeguard participant confidentiality, prior to the start of the focus group sessions, each participant blindly selected a name tag to use to identify themselves each time they spoke. Topics for the AD focus groups were like those used for the previous SN and AT focus groups and included support for the law, implementation problem areas, impact on workload, and recommendations. Each session lasted approximately one and a half hours.

This project was reviewed by the Institutional Review Boards at MDPH and Boston Medical Center.

Data collection and analysis

To collect data on demographics, a short questionnaire was completed by participants at the beginning of each focus group session. This questionnaire asked ADs about: (a) region of the state where their school was located; (b) whether their school served an urban, suburban or rural area; (c) grades included by their schools (Pre-K, elementary, middle and/or high school); (d) whether their school was public or private; (e) how many full-time ADs were employed at their school; and, (f) if their school or school district employed a full- or part-time licensed AT. This questionnaire was handed out at the beginning of the focus group session, completed anonymously and placed in a provided envelope, sealed by the participant, and collected by the focus group facilitator. Data are presented in aggregate form.

Two investigators (JH, KO) listened independently to the audiotape recordings of each focus group and took notes. These notes were then combined with notes taken at the focus groups. These investigators independently prepared, for each focus group, a list of salient points and a narrative summary of the findings deemed most important. They then met to reconcile their lists of salient points and summaries. 

## Results

Participants

Eleven ADs participated in the first focus group and four participated in the second. Fourteen ADs completed the demographics questionnaire, one did not. Six participants were from Central MA, one from Northeast MA, three from Metro Boston, two from Cape Cod and Islands, and two from Western MA. Five participants indicated that their schools served an urban population, six a suburban population, and three a rural population. Nine of the ADs worked with high school students, three with high school and middle school students, and two with schools that spanned grades from Pre-K through high school. Eleven ADs worked at public schools, one at a private school, and one at vocational/technical schools. Ten ADs indicated that their schools employed one full-time AD and four indicated that their schools employed part-time ADs. Eight reported that there was an AT in their school/district and six reported that there was no AT. Of the eight ADs that had an AT in their school/district, five reported that their AT was full-time and three reported their AT was part-time. Participant characteristics are shown in Table 1.

**Table 1:** Characteristic of focus group participants (N = 14)

Region	
Central	6/14 (43%)
Northeast	1/14 (7%)
Metro Boston	3/14 (21%)
Cape and Islands	2/14 (14%)
Western	2/14 (14%)
Urban/Rural	
Urban	5/14 (36%)
Suburban	6/14 (43%)
Rural	3/14 (21%)
School Age Range	
High School	9/14 (64%)
Middle School	0/14 (0%)
Elementary	0/14 (0%)
Pre-K-High School	2/14 (14%)
Middle & High School	3/14 (21%)
School Type	
Public	11/14 (79%)
Private	2/14 (14%)
Vocational-Technical	1/14 (7%)
How Many Full-Time Athletic Directors at School?	
0	4/14 (27%)
1	10/14 (42%)
Athletic Trainer at School?	
Yes	8/14 (57%)
No	6/14 (43%)
Full-Time or Part-Time Athletic Trainer?	
Full-Time	5/8 (63%)
Part-Time	3/8 (38%)

Summary of findings

Support for the Law

The results of the focus groups suggest that ADs generally support the state’s concussion law and regulations. While ADs found some aspects of implementation burdensome, there appeared to be a consensus among the focus group participants that the law was necessary and important for protecting student athletes:

“[It] needs to be in place, but it is cumbersome.”

“[It has] been helpful and necessary, frustrations are keeping up with changes in regulations and how much information is out there.”

“The law is necessary but there is confusion between the parties involved, for example, are the doctor notes actually written by the parents who want their kids to play, etc.”

ADs also indicated that school administrators supported the law, but some suggested that principals and superintendents had little involvement with implementation, placing that responsibility on staff:

“The support above is always there because liability falls on me”

“…but you also feel they’re passing down the responsibility, they don’t know regulations/protocols”

“They have no clue what’s going on”

“Say they’ll help no matter what but won’t help financially.”

Implementation Problem Areas: Problems with Health Care Providers 

AD’s often reported difficulties interfacing with students’ healthcare providers relative to return-to-activity following a concussion. They cited inconsistent care practices and premature and prospective medical clearance, without regard to return-to-play guidelines:

“The pediatrician clears them in one week and they’re good to go, but the trainer states they haven’t gone through the 7 steps.”

Participants explained how the discordance between physician practice and regulation protocols led to confusion, especially among students and parents. Participants felt that requirements for physician training needed to be enforced by MDPH and that other school staff, such as teachers and guidance counselors, should also be required to have concussion training:

“Difficult because we had to tell doctors what to do, they had no idea about protocols”

“Physicians weren’t really educated about protocols.”

Moreover, some ADs expressed discomfort when conflicts with physicians arise over return-to-play procedures:

“Frustrating when the state has return-to-play [RTP] protocol, but doctor gives out UNC [University of North Carolina] RTP protocol, which is different - better to follow doctor rather than school?”

 “Plans don’t match up between state regulations and what doctors are handing out.”

Implementation Problem Areas: Problems with Parents and Students

ADs described some challenges associated with parents and students that are resistant to following school return-to-play protocols. Specifically, they described how some parents prioritize their child’s return-to-play over safeguarding against potential safety risks that come with returning to play too soon after mTBI:

“Adversary-like relationship with parents about forms, why their children cannot play, etc.”

“[Parents] want their child to get a D1 scholarship, no matter what”

“If parents don’t like that the child’s physician does not clear them to play, they’ll find another doctor that will… weird position to be in.”

Additionally, ADs indicated some parents and students were resistant to following school return-to-activity policies. ADs described ways in which some students attempt to subvert regulations by using return-to-activity protocols to avoid schoolwork and tests by feigning symptoms, or alternatively, by denying symptoms to accelerate return-to-play:

“Kids are always trying to beat the system, really frustrating because it cuffs coaches and they can’t question the kids’ symptoms”

“At MCAS time, every kid has a concussion.”

Implementation Problem Areas: Problems with Information Flow

ADs described significant barriers to effective communication and information flow among concussion management team stakeholders, including SNs, guidance counselors, teachers, and other school staff. Some of these communication problems resulted from the fact that many SNs and other staff leave at the end of the teaching day, just as the ADs are arriving for extracurricular athletics:

“The ultimate goal is necessary to make sure people are aware of the important implications of concussions, but there are so many moving parts so it’s a difficult task to bring [a] simplified form of information to all parties involved [parents, players, coaches].”

Another manifestation of lack of information flow is that ADs are sometimes not aware of mTBIs that occur in non-extracurricular sports (e.g. community sports leagues, weekend activities, skateboard or traffic crashes):

“For example, a kid could have gotten injured at band practice or drama practice, and the teachers and athletic directors don’t know and don’t know enough information to put the kid through the regulations”

“Come across times when parents don’t communicate their child was hurt in backyard and received concussion that way, and if they do communicate that to school, it has been as much as a week later. School needs to know ASAP”

“Have regulations nailed down in athletic field, but when it happens outside of athletics, it’s difficult to communicate”

“Child got hurt in band practice over the weekend, but no one told the school so we didn’t know. Frustrating when you’re trying to do the right thing, but the concussions aren’t being reported to you.”

It was noted that the Health Insurance Portability and Accountability Act (HIPAA) regulations were a constraint on information flow about mTBI that occurred in venues other than school athletics.

“HIPAA rules restrict us from supporting our nurses, coaches, athletic trainers, etc. which leads into physical piece but also concussion piece”

“If we can’t get physical reports in, we’re not getting concussion reports in. And if we are, the concussion protocol is not being followed.”

This lack of information flow makes it difficult for ADs to know whether or not a child should be participating in extracurricular sports, and therefore compromises the ability to follow return-to-play protocols specified by the regulations. Despite challenges associated with communication among the school stakeholders, one participant said that at his school, ADs, SNs, and guidance counselors had a concussion management team that meets to review current cases:

“The biggest thing that has been helpful is having a ‘concussion team’ which includes the athletic director, school nurse, guidance counselor, etc. Meet 2-3 times per year to discuss different scenarios that could occur. We’ve gotten our policy out there, we’ve stuck with it and very strict with it, which has helped us. Team has been extremely helpful and has helped with very difficult cases.”

Another participant suggested that SNs coordinate information flow on students’ mTBI:

“All information should go through school nurse first”

 “I depend on my nurse, so when there is any injury report from doctor, I get a copy of it and go through concussion search."

Impact on Workload

Finally, ADs discussed challenges as they related to their scope of work and expertise. ADs are not healthcare providers, and while the safety of student athletes is among their primary concerns, ADs have not necessarily had medical training in mTBI and the management thereof:

“Teachers need to understand the Athletic Director is not the doctor and they do not have all the answers they are asking regarding the child’s [return to learn] plan.”

Thus, for some ADs, implementation of the laws was perceived as additional and uncompensated work and responsibility in an area in which ADs have limited expertise:

“It’s an unfunded mandate”

“Adds considerable amount of work”

“Trying to do everything the district says is overwhelming and it’s frustrating”

 “The amount of hours to fulfill requirements with paperwork is a lot to ask and there is no money involved”

“Everyone has the right idea to protect their kids from concussion damage, but they’re not paid a lot already and these regulations add more hassle”

“Want to make sure everyone is safe, but adds a lot more work.”

Recommendations

Although the ADs generally supported the law and acknowledged the importance of mTBI as a public health issue, some expressed reservations about the level of public education and understanding of the risks involved:

“Could use support from state to get proper information out about concussions.”

They suggested that because the law focused on sports-related mTBI, parents did not realize that mTBI could result from a variety of causes, in a variety of venues:

“The fear is overtaking the facts, we need information from the researchers and to get that information out there”

“A kid who plays high school football will not get CTE, CTE is more common in drug users than football players but that information is not being told to public”

“Information overload, there’s so much information on concussions but it’s easy to get lost in information”

“How we present concussions is important i.e. it’s not just from football, but cheerleading, snowboarding”

“Negative publicity and is hurting athletics.”

## Discussion

Overall there was a high degree of concordance across the three professions (SNs, ATs, ADs) with whom we have conducted focus groups thus far, especially regarding the importance of the law. ADs were more likely than SNs and ATs to experience the implementation of the law as burdensome, although most issues were related to communications among stakeholders. ADs were similar to their SN and AT colleagues in reporting difficulties interfacing with students’ healthcare providers relative to return-to-activity following mTBI. As was the case with SNs and ATs, ADs generally felt supported by school administrators, but spoke of difficulties with parents who were resistant to following school return-to-play policies. Moreover, SNs, ATs, and ADs all noted that some students “game” the regulations, using return-to-activity protocols to avoid schoolwork and tests by feigning symptoms, or alternatively, by denying mTBI symptoms to accelerate return-to-play. Finally, ADs also agreed with assessments by SNs and ATs that teachers, guidance counselors, and healthcare providers could use more training regarding mTBI, and that MDPH should develop training programs for school personnel, and enforce training requirements for physicians. Notwithstanding the overlap in the AD’s, SN’s, and AT’s perceptions of the law, there were differences in their experience of implementation. Relative to their colleagues, ADs spoke more frequently of the burden of implementing the law in terms of time and responsibilities.

Sports concussion laws vary from state to state, and not all require ADs to be involved in the reporting and management of youth sports-related mTBI. In 2016, Doucette et al. conducted qualitative research examining the challenges of implementation of sports concussion legislation among stakeholders in MA [10]. Our findings are consistent with those of Doucette and colleagues, who found that ADs report challenges in terms communicating with physicians and physicians’ lack of training and education about sports concussion law and regulations [10].

Establishing a concussion management team (CMT) is considered best practice for monitoring a student’s recovery and ensuring compliance with state regulations. CMTs should consist of at least the school’s AD, SN, and guidance counselor, but might also include teachers, student physicians, coaches, students, and parents [11]. If establishment of a CMT is too resource intensive, schools might consider electing a point person to advocate for students’ needs. The CMT should meet frequently following a student’s mTBI and regularly throughout the school year to discuss student cases, develop and implement school-specific best practices, and facilitate efficient information flow and collaboration among stakeholders [11].

Limitations

Observations based on these qualitative focus groups cannot be interpreted as representative of all ADs in MA. Our findings will be used to generate hypotheses that warrant further study using quantitative methods. The evaluation team will work with the focus group findings to develop a survey of ADs about the implementation of the sports concussion law and regulations.

## Conclusions

Our findings suggest that ADs assume a critical role in the management of middle and high school students’ mTBI when given the authority to do so through legislation and regulation. Nonetheless, challenges to the daily application of legislated protocols persist. Increased training and awareness targeting parents and physicians may enhance effective teamwork among relevant stakeholders and mitigate frustrations reported by SNs, ATs, and ADs. School policies that require parents to report head injuries incurred outside of school would help ensure that ADs, ATs and SNs have the information they need to effectively protect students from further injury during extracurricular activities. Implementation of sports-related mTBI protocols should continue to be evaluated. More research is needed to quantify ADs’ perceptions and adherence to the MA sports concussion laws. Results from future analyses can be used to refine best practices that enhance ADs’ and other stakeholders’ abilities to successfully comply with state legislation and advocate for the health and well-being of students. 
